# Case Report: Identification of Two Rare Fusions, *PDK1-ALK* and *STRN-ALK*, That Coexist in a Lung Adenocarcinoma Patient and the Response to Alectinib

**DOI:** 10.3389/fonc.2021.722843

**Published:** 2021-08-13

**Authors:** Hao Zeng, Yalun Li, Ye Wang, Meijuan Huang, Yan Zhang, Panwen Tian, Weimin Li

**Affiliations:** ^1^Department of Respiratory and Critical Care Medicine, West China Hospital, West China School of Medicine, Sichuan University, Chengdu, China; ^2^Department of Respiratory and Critical Care Medicine, Lung Cancer Treatment Center, West China Hospital, Sichuan University, Chengdu, China; ^3^Department of Thoracic Oncology, Cancer Center, West China Hospital, Sichuan University, Chengdu, China; ^4^Department of Respiratory and Critical Care Medicine, West China Hospital, Sichuan University, Chengdu, China

**Keywords:** alectinib, *ALK* fusion, lung adenocarcinoma, targeted therapy, double-fusion variant

## Abstract

Several double *ALK* fusions coexisting in one patient have been reported. However, few studies have reported the clinical efficacy of ALK inhibitors in rare double *ALK* fusions. Here, we described a rare *PDK1-ALK*, *STRN-ALK* double-fusion variant in a patient with metastatic lung adenocarcinoma. The patient responded well to alectinib (600 mg) twice daily. This case shows a promising treatment option for patients with rare *ALK* double-fusion variants.

## Introduction

Anaplastic lymphoma kinase-positive (*ALK*-positive) disease occurs in approximately 5% of all patients with non-small cell lung cancer (NSCLC) (1). It has been reported that in a series of 80 *ALK* fusion-positive patients, 16.2% harbored more than 1 *ALK* fusion (2). More than 20 fusion partners for *ALK* in NSCLC have been reported with the increased utilization of next-generation sequencing (NGS). Several *ALK* double-fusion variants, such as *DYSF-ALK*/*ITGAV-ALK* and *EML4-ALK*/*BIRC6-ALK*, showed a good response to different ALK inhibitors, such as crizotinib and alectinib, respectively (3, 4). The clinical response to ALK inhibitors varies for different *ALK* variants. Alectinib, a highly selective ALK inhibitor, showed superior efficacy and lower toxicity in untreated *ALK*-positive NSCLC than crizotinib. Meanwhile alectinib has also demonstrated an overall survival (OS) benefit in the ALEX study (5, 6). However, few data have reported the sensitivity of alectinib in NSCLC harboring *ALK* double-fusion variants (4). Here, we first present a patient with rare double-fusion variants, *PDK1-ALK* and *STRN-ALK*, who responded well to standard doses of alectinib treatment.

## Case Presentation

**Figure 1:**
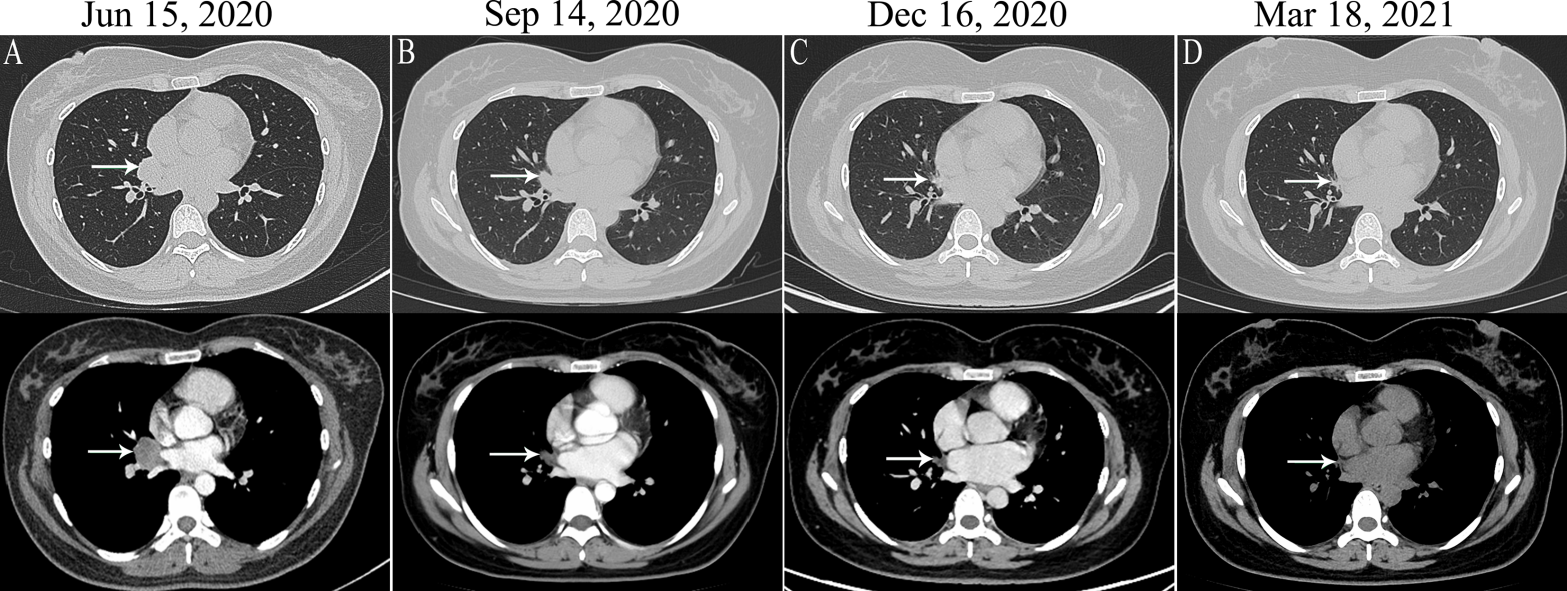
The lung target lesion on CT (white arrow). **(A)** Computed tomography (CT) scan showed a 2.7 cm×2.5 cm mass in the hilum of the right lung before treatment. **(B)** the imaging of lung target lesion after 1 month of alectinib treatment (1.4 cm × 1.1 cm lesions). **(C)** the imaging of lung target lesion after 4 months of alectinib treatment (1.2 cm × 1.0 cm lesions); **(D)** the imaging of lung target lesion after 7 months of alectinib treatment (1.1 cm × 0.7 cm lesions).

**Figure 2:**
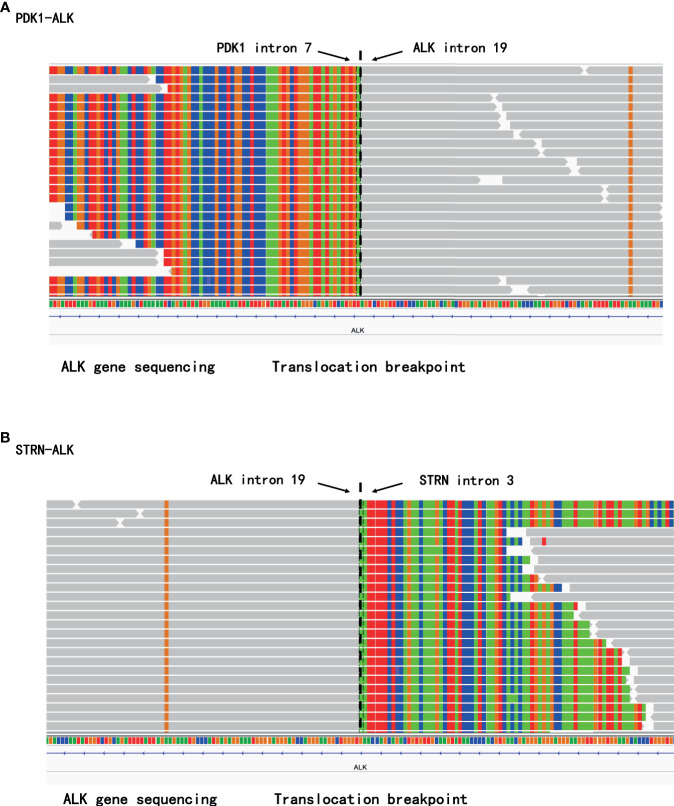
The rare *PDK1-ALK* and *STRN-ALK* fusion was identified in the same tumor tissue by next-generation sequencing (NGS). **(A)** sequencing reads of *PDK1* and *ALK* by the Integrative Genomics Viewer. **(B)** sequencing reads of *STRN* and *ALK* by the Integrative Genomics Viewer.

A 29-year-old Chinese female non-smoker presented to our hospital with a 2-month history of chest pain. She was an accountant without a family history of cancers. She did not have a history of exposure to any professional or environmental carcinogen. Superficial lymph nodes were not palpable, and no other physical examinations showed abnormalities. On June 15, 2020, a contrast-enhanced computed tomography (CT) scan showed a 2.7 cm×2.5 cm mass in the hilum of the middle lobe of the right lung (**Figure 1A**) with mediastinal lymph node metastases and multiple metastases in the ribs and thoracic vertebras. Transthoracic needle biopsy established the pathologic diagnosis of lung solid-predominant adenocarcinoma (T2N2M1c, stage IVB). ALK-Ventana (D5F3), keratin 7, napsin A and thyroid transcription factor-1 were positive by immunohistochemistry staining, and then *PDK1-ALK* (P7:A20, allelic frequency: 12.85%), *STRN-ALK* (S3:A20, allelic frequency: 27.11%) double-fusion (**Figures 2A, B**) and a variable splicing mutation in exon 9 of *TP53* (allelic frequency: 4.84%) were identified in the same tumor tissue by DNA-based NGS (mean depth of the reads was 1200X), while the other driver gene mutations, including *EGFR*, *KRAS* and *ROS1*, were not detected. Given the promising efficacy and tolerability of alectinib, the patient was administered alectinib (600 mg twice daily) as the first-line treatment. A follow-up CT scan found that the lesions shrank to 1.4 cm × 1.1 cm after 1 month, 1.2 cm × 1.0 cm after 4 months, and 1.1 cm × 0.7 cm after 7 months of alectinib treatment (**Figures 1B–D**). The response of the reduction of the mass size changes with the continue of the treatment. The patient is still in follow-up, and no severe adverse reactions have been observed.

## Discussion

Many partners for *ALK* fusions in NSCLC have been reported, including the Huntington interacting protein 1 gene (*HIP1*), baculoviral IAP repeat containing 6 gene (*BIRC6*), and B cell CLL/lymphoma 11A gene (*BCL11A*), which benefit from crizotinib therapy (7–9). Alectinib, ceritinib and brigatinib are currently recommended as first-line choices in Europe and lorlatinib also in the USA (5, 10, 11). Our case first describes the promising efficacy of alectinib treatment of two rare fusions, *PDK1-ALK* and *STRN-ALK*, in a patient with lung adenocarcinoma.

The pyruvate dehydrogenase kinase 1 (*PDK1*) gene is located on chromosome 2q31.1 and has 24 exons, and is a direct hypoxia-inducible factor 1 (HIF1) target gene. Hypoxia, a universal feature of solid tumors, induces *PDK1* gene expression caused *via* HIF1 (12). The expression levels of PDK-1 mRNA and protein were markedly elevated in NSCLC, and knockdown of *PDK-1* can induce cancer cell apoptosis through the Hippo-YAP/IRS2 signaling pathway (13). To date, no other *PDK1* fusion gene has been reported. Our patient harbored a *PDK1-ALK* fusion with intron 7 of *PDK1* ligated to intron 19 of *ALK*, and experienced clinical improvement with alectinib treatment.

Similarly located on chromosome 2, the *STRN* gene encodes a protein with a coiled-coil domain that leads to constitutive activation of ALK kinase *via* dimerization (14). *STRN-ALK* has been reported in three cases of lung adenocarcinoma that were administered ALK inhibitors, and two showed a response to crizotinib and ceritinib. However, a 51-year-old Japanese male who was diagnosed with recurrent lung adenocarcinoma harboring *STRN-ALK* was resistant to alectinib 600 mg once a day as first-line therapy (**Table 1**) (14–16). Nakanishi Y et al. (16) attributed the nonresponse of alectinib to vimentin expression and ATP binding cassette subfamily B member 1 (ABCB1) mRNA overexpression. Additionally, pharmacokinetic data indicated that doses of alectinib were related to clinical activity in a dose-escalation study (300-900 mg twice a day) (17), and Gadgeel et al. (17) chose 600 mg alectinib twice a day as the recommended dose. Similarly, even in an Asian patient population, Zhou et al. (18) confirmed the clinical benefit of alectinib (600 mg twice daily) as a first-line treatment for *ALK*-positive NSCLC. The use of a low dose of alectinib might be a potential cause of treatment failure in patients who came from Japan.

**Table 1:** *STRN-ALK* fusion in patients with lung cancer in previous studies.

References	Age (y), Sex	Type of Tumor	Stage	ALK Inhibitors	PFS (mos)	OS (mos)
Ren H et al. (14)	52, female	LAC	IV	Crizotinib (first-line)	48	
				Ceritinib (second-line)	24	>72
Yang Y et al. (15)	59, male	LAC	IV	Crizotinib (third-line)	29	36
Nakanishi Y et al. (16)	51, male	LAC	IV	Alectinib (first-line)	3	6

LAC, lung adenocarcinoma; PFS, progression free survival; OS, overall survival.

A few limitations are associated with the study. Due to the rarity of the *ALK* double-fusion variants, only one patient was reported in our case. The results need more cases or large cohort studies to verify in the future.

## Patient Perspective

The patient thought that we diagnosed the disease promptly and treated appropriately, and she would continually follow doctors’ advice.

## Conclusions

We are first to report two novel rare fusions, *PDK1-ALK* and *STRN-ALK*, that coexist in one patient with lung adenocarcinoma and are sensitive to alectinib. These double *ALK* fusions responded well to treatment with a standard dose of alectinib. Additionally, NGS assays can provide reliable diagnostic information on novel fusion partner genes for patients with NSCLC.

## Data Availability Statement

The datasets presented in this study can be found in online repositories. The names of the repository/repositories and accession number(s) can be found below: http://www.biosino.org/node/project/detail/OEP002216, We have uploaded detailed raw data of sequences to https://www.biosino.org/node/. All data can be viewed in NODE (http://www.biosino.org/node) by pasting the accession (OEP002216) into the text search box or through the URL: http://www.biosino.org/node/project/detail/OEP002216.

## Ethics Statement

The patient involved in this case report provided written informed consent authorizing the use and disclosure of her protected health information. Written informed consent was obtained from the patient in accordance with the Declaration of Helsinki for publication of the clinical data and any accompanying images. Institutional approval was not required to publish the case details. The patients/participants provided their written informed consent to participate in this study. Written informed consent was obtained from the patient for publication of the case report and the accompanying images.

## Author Contributions

Conceptualization: HZ, Y-LL, YW, M-JH and YZ. Data collection: HZ, Y-LL, YW, M-JH and YZ. Writing-original draft preparation: HZ and Y-LL. Administrative Support: P-WT and W-ML. All authors contributed to the article and approved the submitted version.

## Funding

This work was supported by the National Science Foundation of China (grant number 82072598, 81871890, and 91859203); the National Key Development Plan for Precision Medicine Research of China (grant number 2017YFC0910004); the Science and Technology Program of Sichuan, China (grant number 2020YFS0572) and the Major Science and Technology Innovation Project of Chengdu City, China (grant number 2020-YF08-00080-GX).

## Conflict of Interest

The authors declare that the research was conducted in the absence of any commercial or financial relationships that could be construed as a potential conflict of interest.

## Publisher’s Note

All claims expressed in this article are solely those of the authors and do not necessarily represent those of their affiliated organizations, or those of the publisher, the editors and the reviewers. Any product that may be evaluated in this article, or claim that may be made by its manufacturer, is not guaranteed or endorsed by the publisher.

## Abbreviations

NGS, next-generation sequencing; NSCLC, Non-small cell lung cancer; CT, computed tomography; HIP1, huntington interacting protein 1 gene; BIRC6, baculoviral IAP repeat containing 6 gene; BCL11A, B cell CLL/lymphoma 11A gene; PDK1, pyruvate dehydrogenase kinase 1; HIF1, hypoxia-inducible factor 1; ABCB1, ATP binding cassette subfamily B member 1; LAC, lung adenocarcinoma; PFS, progression free survival; OS, overall survival; CK7, keratin 7; TTF-1, thyroid transcription factor-1; EGFR, epidermal growth factor receptor; KRAS, Kirsten rat sarcoma viral oncogene homolog.
